# PReS-FINAL-2068: S100 A8/A9 serum levels reflect disease activity in juvenile idiopathic arthritis-associated uveitis

**DOI:** 10.1186/1546-0096-11-S2-P80

**Published:** 2013-12-05

**Authors:** K Walscheid, D Holzinger, C Heinz, C Arnold, D Bauer, M Busch, M Hennig, S Wasmuth, J Roth, A Heiligenhaus, D Foell

**Affiliations:** 1 Department of Ophthalmology, St. Franziskus-Hospital Muenster, Germany; 2 Department of Pediatric Rheumatology and Immunology, University Hospital Muenster, Germany; 3 Ophtha-Lab, Department of Ophthalmology, St. Franziskus-Hospital Muenster, Germany; 4 Institute of Immunology, University of Muenster, Muenster, Germany

## Introduction

Juvenile idiopathic arthritis (JIA) is the most common disease associated with uveitis in childhood. S100 proteins have been proven to be valuable biomarkers in different subtypes of JIA as well as in other (auto)inflammatory or autoimmune diseases.

## Objectives

This study analyzes the levels of S100 A8/A9 complexes in the sera of children with uveitis of different entities as well as the correlation between S100 A8/A9 levels and clinical course of ocular disease.

## Methods

Serum samples were collected from patients with juvenile idiopathic arthritis-associated uveitis (JIAU, n = 97), idiopathic anterior uveitis (IAU, n = 26), idiopathic intermediate uveitis (IIU, n = 36), anterior herpes uveitis (n = 3), and healthy controls (n = 10). In 29 patients, aqueous humor was available for analysis. S100 levels were measured by ELISA and were correlated with disease activity as well as other clinical and epidemiological data.

## Results

When compared to healthy controls, elevated S100 A8/A9 serum levels were found in JIAU, IAU and IIU. Preliminary data show an association between uveitis activity and S100 levels. S100 A8/A9 levels correlated with arthritis activity, but independently also with uveitis activity. S100 levels were also increased in aqueous humor from patients with anterior uveitis.

## Conclusion

S100 A8/A9 complexes are a useful biomarker in monitoring articular disease in JIA, but could also be valuable for assessing uveitis. Elevated serum S100 levels could also be observed in patients with idiopathic uveitis without associated systemic disease.

## Disclosure of interest

None declared.

